# Analyzing the impact of COVID-19 on consumption behaviors through recession and recovery patterns

**DOI:** 10.1038/s41598-024-51215-3

**Published:** 2024-01-19

**Authors:** Rui Chen, Tong Li, Yong Li

**Affiliations:** Department of Electronic Engineering, Tsinghua University, Beijing, 100084 China

**Keywords:** Health care, Computational science, Scientific data

## Abstract

The COVID-19 outbreak has dramatically impacted the economy, particularly consumption behaviors. Studies on how consumption responses to COVID-19 can be a powerful aid for urban consumption recovery. In this paper, based on a high-frequency consumption dataset from January 6, 2020, to April 28, 2020 covering 18 sectors and dataset from the corresponding lunar period in 2021, we look at how COVID-19 changed how people spent their money by looking at patterns of recession and recovery during the pandemic. Specifically, we first explore the recession-recovery pattern of national consumption and the effects of various policies and quantify it using regression methods. Then, recession-recovery patterns across cities are widely studied. We also reveal how consumption structures change during a pandemic and the relationship between patterns of change in citizens’ consumption and the socioeconomic characteristics of cities. And the specific empirical analysis is provided through panel regression models. In general, national consumption represented a Vshaped pattern during the pandemic, experiencing a dramatic decline and a rapid rebound. Consumption is significantly inhibited by lockdown, while it is stimulated positively but gradually by easing policies. Consumption patterns at the city level are associated with socioeconomic characteristics. Cities with high-income groups experience a more significant decline, and cities with a high share of the secondary sector have a higher recovery rate in consumption. The consumption structure redistributes but does not fundamentally change. During the recession and early recovery phase, consumption related to basic living saw a significant rise, whereas leisure-related consumption dropped dramatically and recovered slowly. Our study can assist policymakers in implementing diversified market provisions and targeted lockdown policy adjustments for consumption recovery in cities with different socioeconomic backgrounds.

## Introduction

The beginning of COVID-19 in Wuhan in 2020 was described as the biggest ‘black swan’ event to hit China in more than a decade. Incalculable loss of life and property has been caused by the COVID-19 outbreak. To combat this incredibly contagious coronavirus, the Chinese government has successfully implemented preventive measures^1–3^. Policies like city lockdown, home quarantine, travel restrictions, the closure of entertainment venues, and bans on public gatherings were swiftly implemented throughout China within a few days^4–6^. It has been demonstrated that these anti-epidemic measures stop the coronavirus from spreading^6–11^, however, the pandemic and lockdown measures together had a significant negative impact on China’s economy, particularly on citizens’ consumption^12,13^.

Urban consumption is an important driver of economic growth. Meanwhile, urban consumption can be significantly influenced by major economic shocks, such as the Great Recession of 2007–2008, which had a substantial impact on household consumption behavior due to factors like rising home equity and permanent income shocks^14^. Significant external economic shocks may jointly determine income, household consumption, and borrowing dynamics. However, the use of micro-level consumption data helps mitigate this concern. Many researchers have paid close attention to how the COVID-19 outbreak affects consumption. Studies on the consumption responses to COVID-19 pandemic are rapidly emerging^15–19^. Previous studies have examined the pandemic impacted consumption behaviors from multiple perspectives, including impacts of the COVID-19 and lockdown policies^20–23^, changes in consumption structure^21–24^, social-economic factors affecting consumption during COVID-19 pandemic^25–27^. Some have studied the direct impact of COVID-19 on consumption and identified that it had a huge negative impact on consumption, resulting in a dramatic drop in consumption volume in various countries and regions around the world^15,20,23,28,29^. Specifically, these studies focused on studying the trend of consumption during the recession phase. For example, the downward trends of state-level consumption and consumption across sectors were widely investigated in various countries or regions like Europe^30^, North America^31–33^ and China^23,29,34^. Some studies have focused on the effects of COVID-19 response policies, as it is highly useful to test intervention effectiveness and offer policy-relevant lessons. They have measured how representative COVID-19 lockdown policies, such as city lockdown, capacity restrictions and social distancing laws, affect consumption behaviors during the pandemic^20,35–37^.

Existing studies only explain how the COVID-19 pandemic causes a drop in consumption behavior^15,20,28,29^, however, it is potentially assumed that there exists a consumption basket whose ingredients do not change over this course. Therefore, the previous research is almost silent on how the consumption structure, the proportion of various consumption expenditures in the total expenditure, changes before and during the pandemic. Additionally, they do not delve into the whole recession-recovery patterns of both consumption expenditure and consumption structure over the course of the pandemic. Many research studied changes in consumption at the national level, whereas our understanding of consumption recession-recovery patterns at the city level is insufficient. The relationship between socioeconomic characteristics at the city level and consumption patterns during recessions and recoveries is also much less well understood. Consumption is an essential part of economic development. For the purpose of identifying the consumer market pattern in the post-COVID period and the development of consumer upgrading among citizens, it is crucial to comprehend changes in consumption during COVID-19. Understanding how the COVID-19 outbreak and response policies impact the consumption behaviors of citizens over the pandemic period is critical to helping design strategies to prevent the potential economic and societal impacts of changing consumption demands. To achieve this goal, extensive and large-scale research that goes beyond simple descriptive analysis is required.

In this study, we investigate the impact of COVID-19 on consumption behaviors by revealing the recession-recovery pattern of consumption at the national and city levels over the pandemic period. We use high-frequency consumption data from Meituan as a proxy for official national consumption statistics, to reveal consumption responses to the COVID-19 outbreak, which has high precision in the temporal dimension. Meituan is one of the largest big data platforms for life services in China, records and stores a large number of personal daily life consumption records generated in the course of national economic life, and provides us with high-frequency consumption data to track the dynamic changes of citizens’ consumption during the pandemic. The consumption data covers the period from January 6, 2020 (pre-lockdown) to April 28, 2020 (post-unblock) in 53 major cities across mainland China. Notably, our intention is not to capture all possible consumption behaviors but to use a proxy measure to detect an important state- and city-level temporal change pattern in citizens’ consumption during the COVID-19 pandemic. Specifically, we investigate the whole recession and recovery patterns of consumption in the whole country and more than 50 Chinese cities during the pandemic. Then we quantify and compare the effects of various COVID-19 response policies, with a focus on the easing policies’ diminishing beneficial effects on consumption recovery. We also reveal the patterns of temporal changes in consumption structure. Furthermore, the relationship between a city’s socioeconomic characteristics and its recession-recovery pattern of consumption is also explored.

The main contributions of our study are as follows. First, furthering our understanding of consumption responses to COVID-19, we find that aggregate national consumption displays an apparent V-shaped pattern of recession and recovery, which is characterized by several phases with distinct features. Second, we assess the differential effects of COVID-19 response policies, especially easing policies during the recovery phase. And we fully quantify the impact of policies on aggregate national consumption and consumption at the city level, which is important for a large country with a large population such as China. The city’s lockdown policy has a significant negative impact on consumption. On the contrary, easing policies have diminishing positive effects on consumption. We show that the resumption of work and production greatly contributes to consumption recovery. Reopening of gathering places has only a weak boost to consumption, while lifting lockdown has the mildest or even non-significant effect on consumption compared with other easing policies. Third, we provide preliminary evidence on the nature of temporal change patterns in consumption across sectors during the pandemic, which gives us insights into how consumption structure changes over the pandemic period. We observe that COVID-19 alters consumption structure in the short term, but without fundamental changes. In particular, we discuss in what sense these changes in consumption reshape sustainable consumption. Last, we further investigate in depth the association between city-level recession-recovery patterns of consumption and socioeconomic attributes, which has not been considered in any previous studies. Specifically, we focus more on the dynamic patterns of different consumption sectors and used the panel regression model to demonstrate the heterogeneous effects of pandemic changes on urban consumption across different socioeconomic backgrounds.

Notably, income significantly influences the recession of city-level consumption, while the economic structure plays a key role in the recovery phase. It suggests that COVID-19 has exacerbated the shortcomings of economic inequalities across social and economic groups.

## Results

### Temporal variations of consumption behaviors

**Figure 1:**
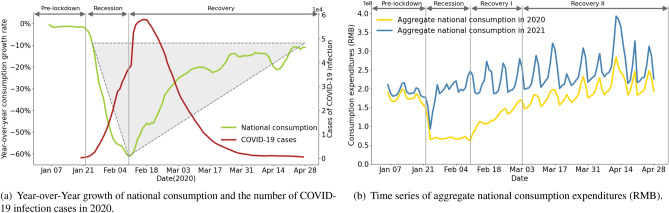
Temporal variations of national consumption behaviors. (**a**) Year-over-Year growth rate of aggregate national consumption and cases of COVID-19 infection. (**b**) Time series of aggregate national consumption expenditure in 2020 and 2021.

Consumption expenditures of citizens exhibit a distinctive V-shaped temporal pattern in terms of aggregate national statistics (Fig.  1a). Notably, the impact of the COVID-19 pandemic on consumption can be analyzed by measuring the change in consumption from two perspectives: the consumption expenditures and the volume of consumption orders. Here, we conduct analysis based on consumption expenditures, and the analysis of the volume of consumption orders is detailed in Supplementary Note 4. With the onset of COVID-19, citizens’ consumption has drastically reduced because of both rising COVID-19 infections and national lockdown policies. Once the pandemic was contained, consumption quickly and significantly recovered. To be specific, national consumption expenditures bottomed out after a sharp decline of about 60% on February 10, 2020. After that, production and work are permitted to resume as the number of COVID-19 cases gradually decreases. As a result, national consumption recovers rapidly. When cities reopen on April 28, 2020, aggregate national consumption recovers by approximately 90%, falling only slightly short of normal levels.

The V-shaped consumption pattern can be separated into three distinct phases, including recession, recovery I and recovery II (Fig.  1b).

- Recession phase (January 23, 2020–February 9, 2020).
  On January 23, 2020, the city lockdown policy was implemented following the COVID-19 outbreak. The drastic reduction in citizens’ consumption behaviors is brought on by store closures and infection-related fear. Notably, the Chinese New Year (January 25) causes consumption spending to decline in both 2020 and 2021. Consumption spending bounces back quickly in 2021 after the holiday, but due to the COVID-19 outbreak, it remains severely depressed (with the lowest level of consumption recorded as − 62%) until February 10 in 2020. Accordingly, year-over-year consumption growth plummets sharply during the recession phase (Fig.  1a), demonstrating the COVID-19 outbreak and lockdown policy have a significant negative impact on residents’ consumption behaviors.
- Recovery phase I (February 10, 2020–March 2, 2020).
  The government declared the resumption of work and production on February 10, 2020, which marks the beginning of recovery phase I. After people gradually resume their regular work and production, their consumption behaviors rebound sharply, creating a V-shaped reversal in national consumption expenditures (Fig.  1a). Notably, consumption expenditure volume is still substantially below normal levels at this phase. Also, cyclical fluctuations, which are visible in consumption temporal variations during normal times, are not present in this phase (Fig.  1b). These show that consumption is still far from returning to normalcy in terms of the expenditure volume and the cyclical pattern.
  Consumption activities for basic needs, including dining, education, and life services, are the main sectors to recover during this phase (Supplementary Note 2). It is interesting to note that hotel consumption has significantly increased, which may be related to the need for workers to undergo a quarantine period before returning to the office. Leisure-related consumption sectors, on the other hand, such as entertainment, karaoke, and travel, remain subdued and do not appear to be on the mend (Supplementary Note 2), where the entertainment sector specifically includes gyms, camping, arcade game centers, massage, etc. The prohibition of group gatherings at this time prevents citizens from engaging in leisure-related consumption behaviors, contributing to the continued depression in related sectors of the economy.
- Recovery phase II (March 3, 2020–April 28, 2020).
  Recovery phase II begins on March 3, 2020, when the government proclaimed that gathering places were once again open. During this phase, national consumption expenditures keep increasing, but at a relatively slower rate than before. By the end of April 2020, national consumption had returned to its pre-epidemic level (Fig. 1a). In this phase, an apparent cyclical fluctuation pattern also appears (Fig. 1b). Both of them imply that citizens’ consumption activities have returned to normalcy at the end of this phase.

The beginning of a leisure-related consumption rebound, including karaoke, entertainment, and travel, can be seen during this phase. In contrast to recovery phase I, this phase is marked by significant cyclical fluctuations in the majority of consumption sectors (Supplementary Note 2). The reopening of gathering places is supposed to encourage residents to engage in more offline consumption, particularly in leisure-related sectors, resulting in a weekend peak in consumption (Fig. 1b).

**Table 1:** Linear regression analysis for aggregate national consumption expenditure.

(a) Regression for YGI		
Linear regression model		
Dependent variable		
Year-over-year growth index (YGI)		
Independent variable	Coefficient	(Standard error)
City lockdown	− 0.55**	(0.08)
Resumption of work and production	0.421***	(0.05)
Reopening of gathering places	0.231***	(0.06)
Lifting lockdown	0.019**	(0.01)
Infected cases of COVID	− 0.418***	(0.07)
Intercept	1.041***	(0.10)
$R^2$	0.792	

(b) Regression for RGI		
Linear regression model		
Dependent variable		
Recovery gap index (RGI)		
Independent variable	Coefficient	(Standard error)
City lockdown	0.489**	(0.09)
Resumption of work and production	− 0.414***	(0.05)
Reopening of gathering places	− 0.115***	(0.03)
Lifting lockdown	− 0.109	(0.02)
Infected cases of COVID	0.382***	(0.06)
Intercept	1.031***	(0.11)
$R^2$	0.727	

***p < 0.01; **p < 0.05.

### Effects of lockdown and its easing

The trend in aggregate national consumption changes dramatically after the lockdown policy is implemented Fig. 1a. Specifically, we can observe that there is a very significant drop in aggregate national consumption after the implementation of lockdown policies (Fig. 1a, January 23, 2020). Correspondingly, after the lifting of restrictions, the aggregate national consumption rebounds immediately (Fig. 1a, February 9, 2020). Clearly, the changes in consumption are associated with the pandemic and intervention policies.

The impact of pandemic response policies on consumption has been observed in relevant literature. Lockdown policies resulted in a sharp decline in consumption, particularly among low-income households^38^. However, the policy effects vary significantly^39^. Therefore, we next investigate how response policies affect economic activity using the first lockdown in China that was imposed in January 2020 and its subsequent gradual easing. To be specific, we perform regression analysis on the year-over-year growth index (YGI) and the recovery gap index (RGI) of consumption activities to quantify the effects. The year-over-year growth index (YGI) is the ratio of the difference in national citizens’ consumption expenditures between 2020 and 2021. The recovery gap index (RGI) is the difference between the national citizens’ consumption expenditures in 2020 and 2021. According to Table 1, the city lockdown policy has a considerable negative effect on consumption activity. Also, COVID-19 infections show strong detrimental effects on the economy, which may be a result of people’s voluntary social distancing in response to rising infections. The lockdown policy shows more harmful impacts on consumption with a larger regression coefficient than COVID infections. Because stores are closed and people are unable to go out, the lockdown restricts both the supply and demand sides of consumption activity. Easing policies, such as the resumption of work and production, the reopening of gathering places, and the lifting of the lockdown, have a positive impact on consumption activity, though their effects are gradually fading. Specifically, consumption greatly benefits from the resumption of work and production, as it has the largest positive coefficients for both indexes. The reopening of gathering places provides a modest boost to consumption. Surprisingly, the coefficients of the lifting lockdown in the two regressions are small or non-significant, indicating that its incentive effect on citizens’ consumption is very limited.

### Recession and recovery comparison between cities

**Figure 2:**
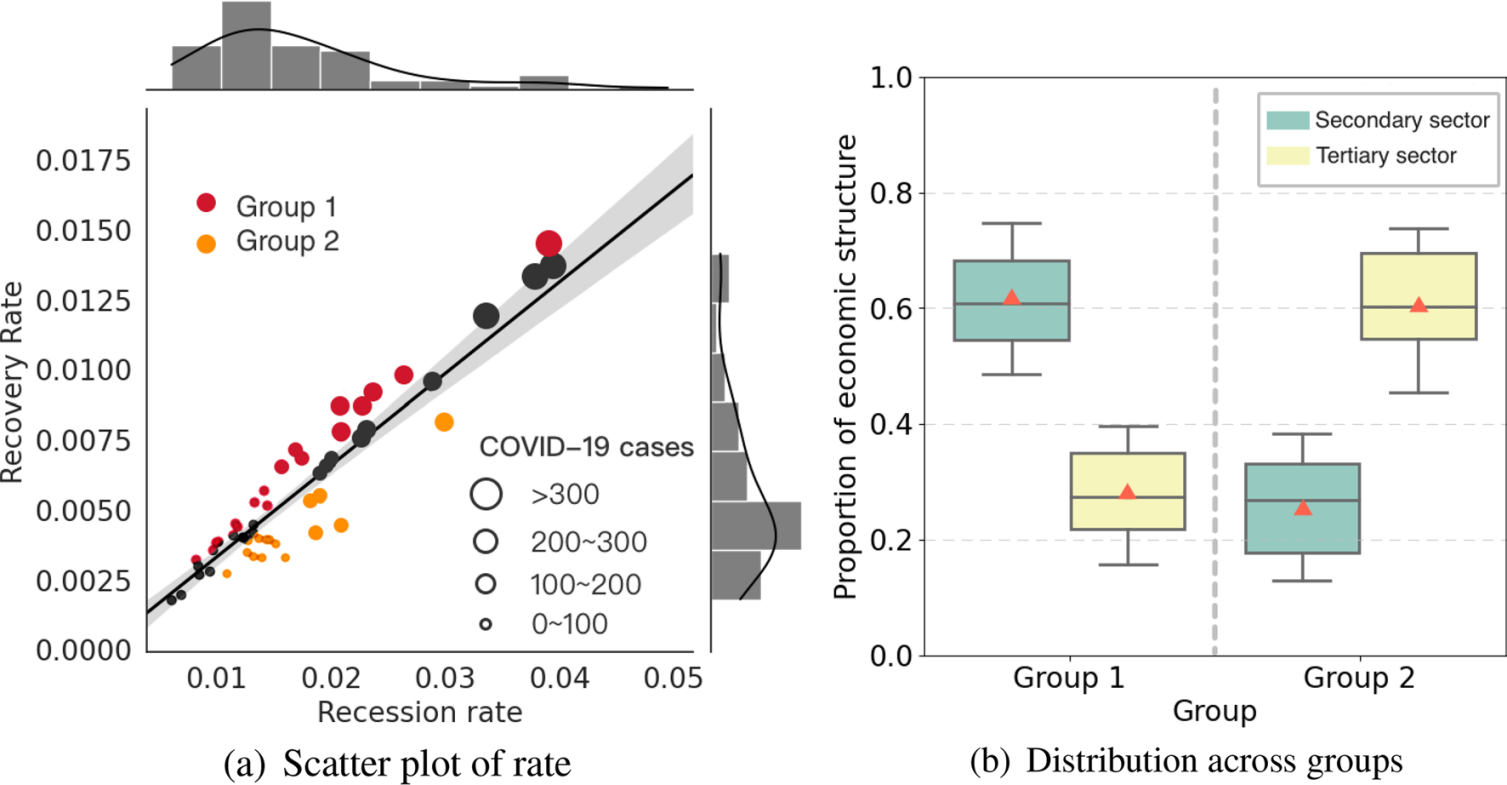
Recession and recovery pattern for 53 cities.

By revealing city-specific recession-recovery rates, we explore how the COVID-19 outbreak influences urban consumption across different cities. The recession rate represents the ratio of the difference in consumption expenditure between the pre-lockdown level and the minimal level to the number of days it took to reach the minimal level. The recovery rate is defined as the ratio of the difference in consumption expenditure between the normalcy level and the minimal level to the number of days needed to return to the normalcy level. The details of the definition of recession and recovery rates are given in Supplementary Note 1. Figure 2a shows the scatter plot of the recession and recovery rates of 53 cities with various degrees of COVID-19 pandemic severity. Cities with more COVID-19-infected cases experienced a significant decline in consumption expenditure within a short time, showing a large recession rate. In general, recession and recovery rates are positively and linearly correlated with one another, corresponding to the V-shaped pattern (Fig.  1a), reflecting a high level of economic resilience in cities.

Based on the relative positions to the regression line, cities can be divided into two groups (Fig. 2a). Cities in group 1, with scatter points above the regression line, have a faster recovery in consumption compared with cities in group 2 (Fig. 2a, Group 1). Alternatively, cities in group 2 with scatter points below the regression line have a lower relative recovery rate (Fig. 2a, Group 2). It is interesting to note that this difference for the two groups of cities is related to one socioeconomic indicator, namely the share of the secondary and tertiary sectors in the city-level economy (Fig. 2b). Specifically, the secondary sector is dominated by the manufacturing and infrastructure industries. The tertiary sector is dominated by service-related industries, such as recreation, socializing, and education (details in Supplementary Note 7). In our case, we focus on the consumption behaviors of citizens and adopt the share of employees in these two sectors to characterize this socioeconomic indicator. Cities in group 1 have a high share ($60\%$ on average) of employees in the secondary sector (Fig. 2b), making them with a developed secondary sector. Cities in group 2 possess a high share of employees in the tertiary sector ($60\%$ on average) (Fig. 2b), which can be classified as cities with a developed tertiary sector. Therefore, the recovery rate observed is associated with differences in city-level economic structure.

**Table 2:** Panel regressions of daily city-level YGI on lockdown and easing policies, COVID-19 cases and social economic attributes.

Panel regression of YGI			
Dependent variable			
Year-over-year growth index (YGI)			
Independent variable	(1)^a^	(2)^b^	(3)^c^
Age between 16 and 59 (labor force)	0.188*** (0.012)	0.234*** (0.013)	0.239*** (0.011)
GDP	0.293*** (0.015)	0.327*** (0.014)	0.325*** (0.016)
Secondary sector	0.392*** (0.020)	0.342*** (0.019)	0.311*** (0.018)
Tertiary sector	0.229*** (0.023)	0.332*** (0.021)	0.237*** (0.022)
Income	0.305*** (0.017)	0.311*** (0.016)	0.329*** (0.015)
City lockdown	− 0.547*** (0.025)	− 0.558*** (0.026)	− 0.549*** (0.027)
Resumption of work and production	0.429*** (0.032)	0.401*** (0.030)	0.403*** (0.029)
Reopening of gathering places	0.238** (0.045)	0.223** (0.043)	0.221** (0.042)
Lifting lockdown	0.069** (0.021)	0.047 (0.020)	0.044 (0.019)
Infected cases of COVID for each city	− 0.073** (0.015)	− 0.092** (0.014)	− 0.103** (0.013)
Nationwide infected cases of COVID	− 0.387*** (0.050)	− 0.399*** (0.048)	− 0.386*** (0.046)
Interaction term			
Age between 16 and 59 (labor force)	N	0.086 (0.010)	0.082 (0.011)
GDP	N	− 0.078** (0.012)	− 0.074** (0.013)
Secondary sector	N	0.281*** (0.014)	0.272*** (0.015)
Tertiary sector	N	− 0.217*** (0.016)	− 0.219*** (0.017)
Income	N	− 0.083*** (0.018)	− 0.085*** (0.019)
City fixed effects	N	N	Y
$R^2$	0.429	0.573	0.616

The table shows point estimates of the regression coefficients for the distinct variables and interaction terms. Standard errors are clustered at the city level.

***p < 0.01; **p < 0.05.

^a^Column(1) gives the basic panel regressions of daily city-level YGI on lockdown and easing policies, COVID-19 cases and socioeconomic variables.

^b^Column(2) add a interaction term between socioeconomic variables with nationwide infected cases of COVID-19.

^c^Column(3) controls for city fixed effects.

**Table 3:** Panel regressions of daily city-level RGI on lockdown and easing policies, COVID-19 cases and social economic attributes.

Panel regression of RGI			
Dependent variable			
Recovery gap index (RGI)			
Independent variable	(1)^a^	(2)^b^	(3)^c^
Age between 16 and 59 (labor force)	− 0.241** (0.010)	− 0.232** (0.009)	− 0.208** (0.011)
GDP	− 0.215*** (0.013)	− 0.298*** (0.014)	− 0.259*** (0.015)
Secondary sector	− 0.221*** (0.018)	− 0.266*** (0.017)	− 0.271*** (0.016)
Tertiary sector	− 0.196*** (0.020)	− 0.249*** (0.022)	− 0.246*** (0.021)
Income	− 0.286*** (0.019)	− 0.396*** (0.018)	− 0.384*** (0.017)
Resumption of work and production	− 0.286*** (0.033)	− 0.396*** (0.034)	− 0.384*** (0.035)
Reopening of gathering places	− 0.174** (0.046)	− 0.135** (0.047)	− 0.127** (0.048)
Lifting lockdown	− 0.016** (0.008)	− 0.013 (0.004)	− 0.008 (0.002)
Infected cases of COVID for each city	0.113** (0.016)	0.104** (0.017)	0.008** (0.001)
Nationwide infected cases of COVID	0.411*** (0.051)	0.423*** (0.052)	0.431*** (0.053)
Interaction term			
Age between 16 and 59 (labor force)	N	− 0.031 (0.010)	− 0.023 (0.011)
GDP	N	0.156** (0.012)	0.162** (0.013)
Secondary sector	N	− 0.004*** (0.000)	− 0.002*** (0.000)
Tertiary sector	N	0.273*** (0.016)	0.249*** (0.017)
Income	N	0.291*** (0.018)	0.281*** (0.019)
City fixed effects	N	N	Y
$R^2$	0.457	0.535	0.596

The table shows point estimates of the regression coefficients for the distinct variables and interaction terms. Standard errors are clustered at the city level.

***p < 0.01; **p < 0.05.

^a^Column(1) gives the basic panel regressions of daily city-level RGI on lockdown and easing policies, COVID-19 cases and socioeconomic variables.

^b^Column(2) add a interaction term between socioeconomic variables with nationwide infected cases of COVID-19.

^c^Column(3) controls for city fixed effects.

We next conduct regression analysis to quantitively reveal how socioeconomic factors affect city-level consumption. Specifically, using a panel regression model (see “Methods” for details), we further quantified the combined effect of city-level socioeconomic variables (labor force, GDP, income, secondary, and tertiary sectors), response policies (the city lockdown and its easing), and daily COVID-19 incidence (nationwide and city-level cases) on the year-over-year growth index (YGI) and the recovery gap index (RGI) of city-level consumption during the period from January 6 to April 28, 2020 and the same period in 2019 (lunar calendar) (Tables 2, 3). The first column gives the basic city-level time series pattern in the data, as a function of all variables mentioned above. It is apparent that city-level consumption is significantly correlated with each variable in the benchmark regression (Tables 2 and 3, column (1)). Cities with a larger labor force, a larger GDP, higher income, or a larger fraction of secondary and tertiary sector workers consume more on average (Tables 2 and 3, column (1)). In columns (2), we included a interaction term between the nationwide COVID-19 incidence and socioeconomic variables to explore how these socioeconomic factors impact city-level consumption, especially during the pandemic (Tables 2 and 3, column (2)). For example, GDP has an interaction term of − 0.078 (Tables 2, column (2)), which implies that the positive impact of GDP on daily city-level YGI continues to weaken as the number of COVID-19 cases increases. In short, the growth of COVID-19 cases weakens the positive impact of GDP on city-level consumption growth. Alternatively, per capita GDP (Tables 2 and 3, column (2)) means that as the number of pandemic cases increases, the positive impact of per capita GDP on total urban consumption continuously diminishes. In such a scenario, it is possible that once the pandemic reaches a certain severity threshold, the positive impact of per capita GDP on total urban consumption weakens to zero or even becomes negative. Therefore, it can be inferred that when the pandemic is below a certain critical value, per capita GDP positively affects aggregate urban consumption. However, when the pandemic exceeds this critical value, per capita GDP may have a negative impact on aggregate urban consumption.

City-level recession-recovery patterns of consumption are associated with socioeconomic attributes during the pandemic. When compared to cities with lower-income groups, cities with higher-income groups see a bigger drop in consumption. Also, cities with a high share of tertiary sector workers recover more slowly in terms of consumption, whereas they recover more rapidly in cities with a high share of secondary sector workers (Tables 2 and 3, column (2)). Such a conclusion persists after additionally controlling for city fixed effects (Tables 2 and 3, column (3)). Moreover, socioeconomic variables have differential impacts on city-level consumption at different phases (Supplementary Note 3). Income and GDP are the most impactful variables in the recession phase, while the economic structure contributes more to the recovery phase. Specifically, the secondary sector counts more in Recovery Phase I, and the tertiary sector is crucial to Recovery Phase II (Supplementary Note 3), which supports our earlier finding about the gradual recovery of leisure-related consumption (Supplementary Note 2).

**Figure 3:**
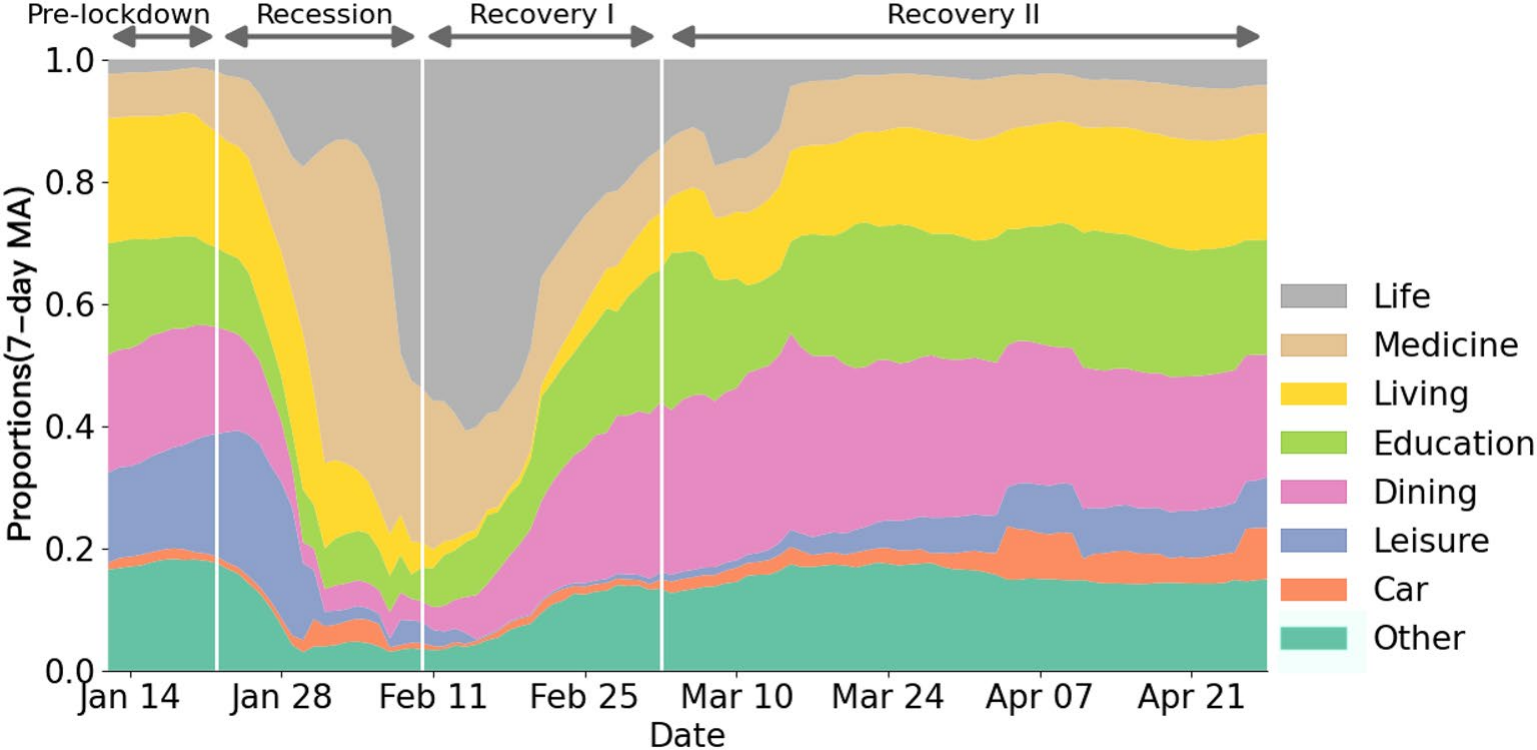
Evolution of the share of aggregate national consumption across sectors during COVID-19 pandemic.

### Consumption structure of citizens during COVID-19 pandemic

The national consumption structure exhibits a remarkable dynamic pattern in alignment with lockdown and easing policies in the recession and recovery phase I (Fig.  3), but eventually recovers to its pre-lockdown pattern. Specifically, consumption structure refers to the share of each type of consumption expenditure in the total consumption expenditure. The details of the definition of consumption structure are given in Supplementary Note 6. Market shares of national consumption across sectors were quite stable prior to the implementation of city lockdown, with each sector distributing roughly even except for a small fraction of life essentials, medicine, and car consumption. Immediately following the city lockdown, a clear pattern of redistribution emerges: spending on life essentials and medicines grows greatly, with these two sectors alone accounting for over 60% of national consumption by early February, while other sectors like education, dining, and leisure shrink entirely, reflecting the sharp growth in demand for daily necessities and health, as well as the collapse in demand for non-essential consumption during the recession phase (Fig.  3). National consumption recovers after the easing of policy restrictions, and the market share of consumption for basic living and working, like dining, education, and car consumption, recovers strongly after the resumption of work and production on February 10, 2020 (Fig.  3). Except for a slow recovery in leisure consumption, the market share of consumption steadily returns to the pre-lockdown distribution in recovery phase II after the reopening of gathering places on March 3, 2020 (Fig. 3).

**Figure 4:**
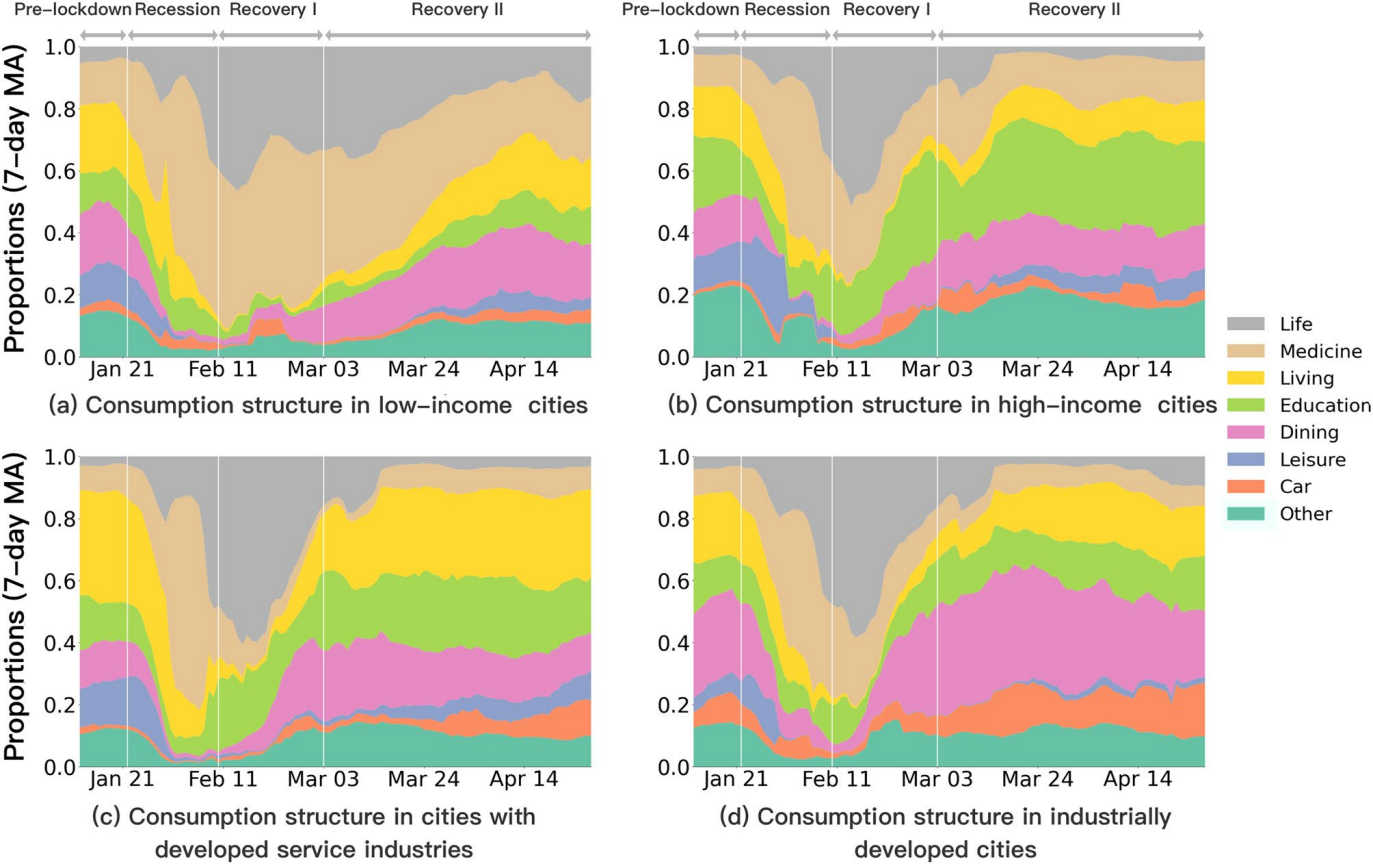
Dynamics of city-level consumption structure patterns during COVID-19 pandemic. Shares are computed as seven-day moving averages (MA).

To investigate the relationship between consumption structure and income levels during the pandemic, we reveal the dynamic consumption patterns of the bottom 10 and top 10 cities ranked by per capita income (Fig. 4a and b). It shows results consistent with previous analysis of national consumption structure (Fig. 3). They exhibit similar dynamic patterns of national consumption structure, but with some remarkable distinctions in terms of market shares. In recession and recovery phase I, the share of medicine and life essentials increase rapidly and significantly in all cities, with other sectors shrinking to varying degrees (Fig. 4a and b). In particular, consumption of education, dining and other (pet and beauty) sectors experience a much faster recovery in high-income cities, when compared with the slow recovery in most consumption, except for medicine and life essentials, in low-income cities (Fig. 4a). Therefore, throughout the entire economic trauma of the pandemic, with medical and daily life-related consumption comprising nearly 80% of the consumption pattern (Fig. 4a), residents in low-income cities emphasized the consumption of daily necessities. Conversely, residents in high-income cities exhibited a faster recovery in consumption across sectors other than life-related and medical consumption (Fig. 4b), with a particular emphasis on education investment (during the recovery period I, education consumption accounts for the second highest share at around 24%) (Fig. 4b) . Notably, during the recovery phase I, their share of education consumption is the highest among all sectors, except for essential daily life-related and medical consumption (Fig. 4b).

By ranking the proportion of secondary and tertiary sectors, we reveal the relationship between dynamics of city-level consumption structure and economic structure (Fig. 4c and d). In recession and recovery phase I, living (hotel and homestay) and education consumption recover faster and account for a large proportion of total consumption in cities with a developed tertiary sector (Fig. 4c), whereas dining and car consumption recover faster and make up a significant proportion of total consumption in industrially developed cities (Fig. 4d). It suggests that citizens place more emphasis on practicality in daily consumption owing to the needs of commuters in cities with a developed secondary sector. Nevertheless, citizens are more development and enjoyment-oriented when they consume in cities with a developed tertiary sector.

## Discussion

Our research concurs, in certain aspects, with the outcomes of preceding studies conducted in China amidst the COVID-19 pandemic. Analogous investigations have noted a swift decline in consumer behaviors subsequent to the enforcement of quarantine measures. Additionally, there was a more pronounced decrease in discretionary spending as compared to essential goods consumption. However, our study diverges from previous works in several pivotal respects. Primarily, we have quantified the role of response policies, discerning a gradual attenuation in the positive effects of policy relaxation on consumption. Secondly, we have meticulously examined, through panel regression models, how socioeconomic factors influence the shifts in consumption behaviors during the pandemic, an area that has not been extensively explored in earlier research. Moreover, by contrasting with former studies, our analysis further reveals that the structural changes in consumption during the pandemic did not fundamentally reverse. We also observed that, notwithstanding the abundance of research on consumer behaviors during the pandemic, studies focusing on how the consumption structure evolves over the course of the pandemic remain relatively scarce. Utilizing high-frequency consumption data provided by Meituan, our study offers a novel perspective to understand this phenomenon.

There are limitations to our study, including the focus on the first wave of the epidemic in China and the need for long-term data analysis. Due to data limitations, we only studied the first wave of the epidemic in China, and more than one year of data analysis may be needed to understand long-term changes in consumption behavior. Additionally, we only studied typical socioeconomic characteristics and did not consider more heterogeneous characteristics at the city level, such as racial differences. Still, our study focuses on tracking dynamic changes in consumption behavior, and future research could further develop predictive methods for consumption behavior prediction.

Our study fills a void in the existing studies on the consumption response to the epidemic by ingeniously demonstrating that the high-resolution data provided by Meituan, a big data platform for life services, can be used as a proxy for national consumption items to analyze the dynamics of consumption during the epidemic. We did a large-scale analysis using different types of real-world data from more than 50 major cities to measure how consumption patterns change over the course of an epidemic’s life cycle and how they relate to socioeconomic factors at the city level. Our results show that government departments can use the data to understand how consumption changed during COVID-19, keep track of changes in the structure of consumption, and change response measures accordingly. Specifically, we find that higher-income groups experience greater consumption declines than lower-income groups during the pandemic. This is likely due to the fact that COVID-19 and lock-down policies, by their very nature, have a greater impact on the enjoyment-focused consumption pattern prevalent among wealthier citizens. With our findings, the government has the opportunity to take radical decisions regarding consumption recovery and addressing inherent inequalities. The provision of more opportunities to support leisure-related consumption, such as travel, movies, and sports, may be crucial to enhancing the openness of our society and economy. Also, it is suggested that the government take steps to create more jobs and ease the unemployment crisis caused by COVID-19. This would raise people’s incomes and encourage them to consume. According to the analysis of the shift in consumption structure during the lockdown, we propose that market supply must be diversified to effectively increase consumer demand. As the change in consumption structure during the pandemic varies across social and economic groups, the market will exhibit characteristics of consumption stratification. In order to achieve a better balance between supply and demand, the market supply must meet the diverse consumption needs of various urban groups. Businesses and service providers in high-income areas are encouraged to increase the number of goods and services that help people grow and have fun. In low-income areas, however, there is an urgent need for goods and services that are affordable and help people live better lives.

## Conclusion

Our research shows how COVID-19 and response policies affect the way people go about their daily lives after a pandemic. First, we discover a V-shaped pattern, as the general consumption recovery is led by a natural increase in consumption without policy-driven effects. After experiencing a sharp decline, consumption rebounded rapidly with the implementation of the resumption of work and production, exhibiting a V-shaped recession-recovery pattern. Second, for the policy response, both the recession and the recovery of consumption are related to the physical constraints imposed by the quarantine. When the quarantine is relaxed, such as with the resumption of work and production, the suppressed consumption for basic needs recovers quickly. We also notice that the positive effect of easing policies on consumption is diminishing. The essence of consumption recovery, which has almost returned to pre-epidemic levels, lies in the strong recovery in consumption for basic needs in the early phase, in contrast to leisure-related consumption, which starts to recover only at a later stage. Third, by comparing recession and recovery patterns of consumption across cities, we reveal that they are associated with differences in city-level socioeconomic attributes like economic structure and citizens’ income. The COVID-19 outbreak has a larger negative impact on consumption recovery for those from cities with developed tertiary sectors. Underlying this result is a slow recovery in leisure-related consumption. On the contrary, cities with developed secondary sectors, often considered to provide more employment and material security, exhibit a faster recovery in consumption. Moreover, we document that higher-income groups suffer larger declines in consumption compared with lower-income groups during the pandemic. Finally, despite the redistribution throughout the lockdown period, there is no fundamental change in consumption structure, which to some extent indicates that consumption is relatively resilient and the trend of consumption upgrading is not reversed by COVID-19.

## Methods

### Data

The vast bulk of our research is based on Meituan’s consumption data in mainland China from 18 different sectors, which consists of daily consumption records. Specifically, the 18 sectors include Entertainment, sports, travel, karaoke, movie, life services, households, shopping, weddings, offspring, hotel, homestay, dining, education, medicine, car, pet and beauty. Meituan platform records consumption data through users’ activities. This includes various user actions such as browsing, searching, ordering, paying, and evaluating on the app. These activities generate a large amount of data, including user behavior, timestamps, geographic location, consumption categories, and other information. Each activity and order is timestamped to record when the user performed an action. To examine the pandemic impact on consumption, we collected consumption records from January 6, 2020, to April 28, 2020, and the data from the same lunar calendar period in 2021 for comparison, thus eliminating the impact of the Chinese Lunar New Year. The dates of January 6 and April 27 are 2 weeks before and 2 weeks after city lockdown, for a total of 112 days, allowing us to analyze the entire COVID-19 period.

Except for movie and karaoke (two sectors that have been missing for a long time due to restriction policies), we filter out cities with inactive consumption by selecting cities with at least one consumption record in each sector within a month. As a result, $72\%$ of all cities in the mainland including 53 cities, are represented in Meituan data.

Due to the sparse format of the data, consumption records for some cities on certain dates may be missing. To solve this problem, we perform data interpolation to recover temporal no-consumption entries. Temporally no record is normal in urban space, but long-term vacancy is likely owing to lockdown policies. If a certain sector of consumption has no record for less than three continuous days, we fill the discontinuous entries with mean values within the gaps. For long-term vacancies that exceed 3 days, we preserve the gaps.

We also collect data on consumption from urban households from the National Bureau of Statistics and the China Urban Statistical Yearbook for comparison and validation. To validate the representativeness of our data, we examine its Pearson correlation with household consumption expenditure data from the China Urban Statistical Yearbook from the temporal and spatial dimensions, which shows a significant correlation, proving that our consumption data can be used as a proxy indicator of official consumption data series (details in Supplementary Note 5).

### Evaluation indicators

We summarize the symbolic representation of variables used in our paper in Table 4. We construct two indicators of year-over-year growth index and recovery gap based on city granularity to further evaluate the recession-recovery pattern of consumption, where *i* represents a city, *t* represents time, and *tc* represents total city-level consumption. The Year-over-Year Growth Index (YGI) is a measure of relative recession and recovery in city-level consumption:

1 $$\begin{aligned} Y G I_{i, t}=\left( t c_{i, t}-t c_{i, t^{\prime }}\right) / t c_{i, t^{\prime }}. \end{aligned}$$

The Recovery Gap Index (RGI) measures the absolute gap in city-level consumption recovery. The Year-over-Year Growth Index cannot reveal the absolute distance of the city returning to normal because it only reflects the relative degree of recovery. Although consumption in some cities is showing signs of improvement, the absolute gap remains large, the distance to full recovery is long, and the risk of a consumption downturn is high. As a result, we utilize the recovery gap as another indicator of city-level consumption recovery:

2 $$\begin{aligned} R G I_{i, t}=t c_{i, t}-t c_{i, t^{\prime }}. \end{aligned}$$

**Table 4:** Symbolic representation of variables.

Variable	Description
$t c_{i, t}$	The total consumption in city *i* in 2020 (from January 6 to April 28)
$t c_{i, t^{\prime }}$	The total consumption in city *i* in 2021(from January 6 to April 28)
$YGI_{i,t}$	Year-over-Year Growth Index in city *i* in 2020, i.e., the percentage change in consumption relative to the baseline year of 2021, compared to consumption during the pandemic outbreak in 2020
$RGI_{i,t}$	Recovery Gap Index in city *i* in 2020
$Y^{(r)}$	YGI or RGI of aggregate national consumption
*X*	Number of infected COVID-19 cases each day across China
$P_j$	Indicator variable for whether a day falls in the post-lockdown period, where *j* can be four separate policies
$\mu _i$	Error term in the linear regression model
$\beta _0$	Intercept in the linear regression model
$x_{i,t}$	Number of infected COVID cases in city *i* on day *t*
$c_t$	Number of infected COVID-19 cases on day *t* across China
$p_j$	Indicator variable that states whether a certain policy is implemented on that day
$X_m,i$	City-level socioeconomic control variables (the percentage of the labor force, the percentage of employees in the secondary sector, the percentage of employees in the tertiary sector, per capita GDP, and per capita income)
$u_i$	City fixed effect that controls for any time-invariant city characteristics
$\varepsilon _{i,t}$	Error term in the panel regression model
$\alpha$	Intercept in the panel regression model

### Empirical strategy

Linear regression modeling is used to study the impact of COVID-19 and its response policises on aggregate national consumption. Linear regression model is a statistical model used to predict the relationship between a dependent variable and independent variables. Its core idea is to minimize the difference between actual and observed values in the dataset so that the difference is greater than or equal to zero. The aggregate national consumption is primarily influenced by lockdown and easing policies and the severity of the pandemic. Therefore, in our study, the independent variables consisted of four different policy variables (all binary) and the number of daily COVID-19 infections (continuous variable). The dependent variable YGI or RGI is a proxy for aggregate national consumption. The purpose of the regression model is to derive the effects of various independent variables on the two consumption indicators according to the results of the regression analysis. The daily YGI and RGI of aggregate national consumption are linearly regressed on lockdown and easing policies. To handle categorical variables, we convert them into dummy variables (also known as indicator variables), where each categorical variable is encoded into multiple binary variables to represent its different categories. This allows us to incorporate policy variables into the model. If China enters a particular phase of the lockdown or lockdown easing on a given calendar day, each lockdown or easing dummy-a binary variable for each period-takes on a value of one and zero otherwise. The reported coefficients can thus be read as the excess percentage point growth of YGI or RGI as a function of COVID-19 cases and the policy adopted at each phase of the pandemic. Taking − 0.418 as an example, for every 1 unit increase in the number of cases, YGI decreases by 0.418 percentage points on average. This means that for each one-unit increase in the independent variable X, the average change in the dependent variable YGI is an increase of 0.418 percentage points. It is important to note that the dependent variable YGI itself is a percentage, so the increase in the average value of YGI as independent variable X increases is measured in percentage points. Let $Y^{(r)}$ be YGI or RGI of aggregate national consumption, and *X* be the number of infected COVID-19 cases each day around China. $P_{j}$ is an indicator variable for whether a day falls in the post-lockdown period, where *j* can be four separate policies. $\mu _{i}$ is the error term and $\beta _{0}$ is an intercept. We perform linear regressions on the following specifications^40^:

3 $$\begin{aligned} Y^{(r)}=\beta _{0}+\beta _{1} X+\beta _{2} P_{j}+\mu _{i}. \end{aligned}$$

To handle categorical variables, we convert them into dummy variables (also known as indicator variables), where each categorical variable is encoded into multiple binary variables to represent its different categories. This allows us to incorporate categorical variables into the model and model their effects. To validate whether the linear regression model is suitable for the dataset, we use the coefficient of determination ($R^2$) to measure the model’s goodness of fit and prediction accuracy

**Table 5:** Explanation of control variables in panel regression.

Variable	Explanation
*Proportion of age between 16 and 59 (labor force)*	The ratio of employed individuals to the total population within a city. This variable provides insights into the city’s employment levels and labor market conditions^41,42^. An increase in the labor force participation rate may lead to increased consumption and stimulate business activity^43^. It is included to control for the potential influence of employment on consumption capacity
*Per capita income*	From the Keynesian consumption function, urban residents’ consumption expenditure is mainly composed of two parts, i.e. spontaneous consumption and induced consumption. The most significant effect on induced consumption is the current disposable income^44^. This control variable accounts for economic disparities among cities, as higher-income cities may have greater disposable income, potentially influencing consumption patterns^45^
*Per capita GDP (Gross Domestic Product)*	Total economic output of a city or region, reflecting its economic size. This is a measure of the economic prosperity of a country or region, reflecting the economic level of an average resident^46^. GDP is included as a control variable to control for the influence of city size on urban consumption. Larger cities tend to have higher GDP, which can affect consumption levels^47^
*Proportion of employees in the secondary sector*	Percentage of individuals employed in industrial and manufacturing sectors within a city. The development of the secondary sector often leads to technological innovation, which affects not only the efficiency of production, but also the supply of consumer goods and services and hence consumer demand^48^. This variable reflects the city’s industrial structure, which may lead to different consumption patterns. It is included to control for the influence of industrial composition
*Proportion of employees in the tertiary sector*	Percentage of individuals employed in the service industry within a city. The sectors with the highest risk of closures and layoffs during the epidemic are the tertiary sector, represented by the accommodation, retail trade, and entertainment and leisure services sectors^49^. This indicator clearly has important implications for the structure of consumer spending as well as the propensity to spend^25^. Like the proportion of employees in the secondary sector variable, this one also reflects the city’s industrial structure and its potential impact on consumption patterns

**Table 6:** Statistics of city-level control variables.

Variable name	Unit	Mean	Standard error	Min.	Max.	Sample size
Proportion of age between 16 and 59 (labor force)	%	52.19	3.6	47.13	59.62	53
Per capita income	10,000 Yuan	4.69	1.46	2.09	7.22	53
Per capita GDP	10,000 Yuan	12.67	2.52	7.8	16.58	53
Proportion of employees in the secondary sector	%	25.85	9.39	11.5	42.97	53
Proportion of employees in the tertiary sector	%	63.25	9.4	46.6	79.23	53

The panel regression model is capable of capturing individual effects that do not vary over time, such as city-level income and the proportion of secondary and tertiary sectors (in Tables 5 and 6) Also, it can observe time-invariant effects that do not vary across cities, such as nationwide infected cases of COVID-19. Socio-economic indicators are considered as control variables at the city level and are typically assumed to remain constant within a specific time frame (from 2019 to 2020). These macroeconomic indicators generally do not experience significant fluctuations over the short term, thus providing a reasonable basis for the assumption of relative stability within this short time period. Here, we use appropriate methods to deal with non-stationarity, such as introducing dummy variables. The dummy variables in the panel data are stationary and do not change over time, so they are not affected by non-stationarity. When using dummy variables in panel regression, the focus is on estimates of fixed effects or fixed-effect models, rather than estimates of time series features. Note that the panel regression model can automatically handle multicollinearity between variables so that the impact of each variable on YGI or RGI can be analyzed independently. In addition, we need to include interaction terms between socioeconomic attributes and COVID-19 cases to test for heterogeneity in the impact of city-level socioeconomic attributes on consumption during COVID-19. Thus, we develop a panel regression model to analyze the interaction between different independent variables and COVID-19 cases in 53 cities in relation to city-level consumption changes. The final formulation of the panel regression model is as follows^50,51^:

4 $$\begin{aligned} y_{i,t}^{(r)}=\alpha +\beta _{0} x_{i,t}+\beta _{1} c_{t}+\beta _{2} p_{j}+\sum _{m=5}^{M} \beta _{m} X_{m,i}+u_{i}+\varepsilon _{i,t}, \end{aligned}$$

where $y_{i,t}$ denotes YGI or RGI in city *i* on day *t*, $x_{i,t}$ is the number of infected COVID cases in city *i* on day *t*, $c_{t}$ is the number of infected COVID-19 cases on day *t* across China, $p_{j}$ is an indicator variable that states whether a certain policy is implemented on that day, *X* represents other independent variables, such as various socioeconomic attributes, and $u_{i}$ is a city fixed effect that controls for any time-invariant city characteristics that might affect disease outcomes or other variables, $\varepsilon _{i,t}$ is the error term, and $\alpha$ is an intercept. All variables are normalized to eliminate the effect of dimensions.

The first column of Table 2 gives the basic city-level time-series pattern for YGI and RGI, as a function of COVID-19, lockdown and easing policies, and socioeconomic attributes. Considering that the impact of COVID-19 on consumption may vary across cities with regard to socioeconomic attributes, to capture this heterogeneity, we include interaction terms for each socioeconomic attribute with COVID-19 in the second column of Table 2, which also improves the fit of both models. In columns (3), we additionally control for city fixed effects to capture unobserved city-specific factors, which provides the best fit.

### Supplementary Information

Supplementary Information.

## Data Availability

Social-economic data is available from the China Urban Statistical Yearbook on the website of the National Bureau of Statistics (https://data.stats.gov.cn). The consumption data that support the findings of this study are available from the Meituan platform. The daily COVID-19 confirmed case for each infected city and the whole country are updated daily by the National Health Commission of China or city-level Health Commissions since January 21, 2020. he datasets used and/or analysed during the current study available from the corresponding author on reasonable request.
