# Supplementary material for: Analyzing the impact of COVID-19 on consumption behaviors through recession and recovery patterns
Source: Sci Rep. 2024 Jan 19;14:1678. doi: 10.1038/s41598-024-51215-3 (PMC10798975; doi:10.1038/s41598-024-51215-3)
Supplement: Supplementary file 1 — Supplementary Information. [file 41598_2024_51215_MOESM1_ESM.pdf]

Supplementary

Supplementary Note 1

Definition of Recession and Recovery Rate

Recession rate: The recession rate is used to measure the rate at which consumption in each city declines from its pre-lockdown level to its minimal level. It is calculated as the ratio of the difference in consumption expenditure between the pre-lockdown level and the minimal level to the number of days it took to reach the minimum. Specifically, for city i, the *recession* rate of consumption is given by the following calculation procedure:

$$recession_i = (pre_i - m_{i,t})/(t_i - t_0), \qquad (5)$$

where m_{it} is the minimum value to which the consumption of city i decreases at day t_i during the lockdown period, and pre_i is the average consumption level of city i before the city lockdown, and t_0 is the date of January 23, 2020 (city lockdown).

Recovery rate: The recovery rate is used to measure the rate at which consumption in each city recovers from its minimal level to its normal level. It is calculated as the ratio of the difference in consumption expenditure between the normal and minimal levels to the number of days it took to reach the normal level. Specifically, for city i, the *recovery* rate of consumption is given by the following calculation procedure:

$$recovery_i = (post_i - m_{i,t})/(t_1 - t_i), \qquad (6)$$

where $post_i$ is the average consumption level of city i after the cities reopen, and t_1 is the date of April 8, 2020 (lifting lockdown).

Supplementary Note 2

Temporal Patterns of Consumption Across Sectors in Each Recovery Phase

To further explore the specific change pattern of various categories of consumption at two recovery phases, we plot the time series of consumption with more importance to daily life, such as dining, and leisure-related consumption, such as entertainment (Supplementary Figures 1 and 2). The former begin to recover after the resumption of work and production on February 10, 2020, while the latter begin to recover only after the opening of gathering places on March 3, 2020.

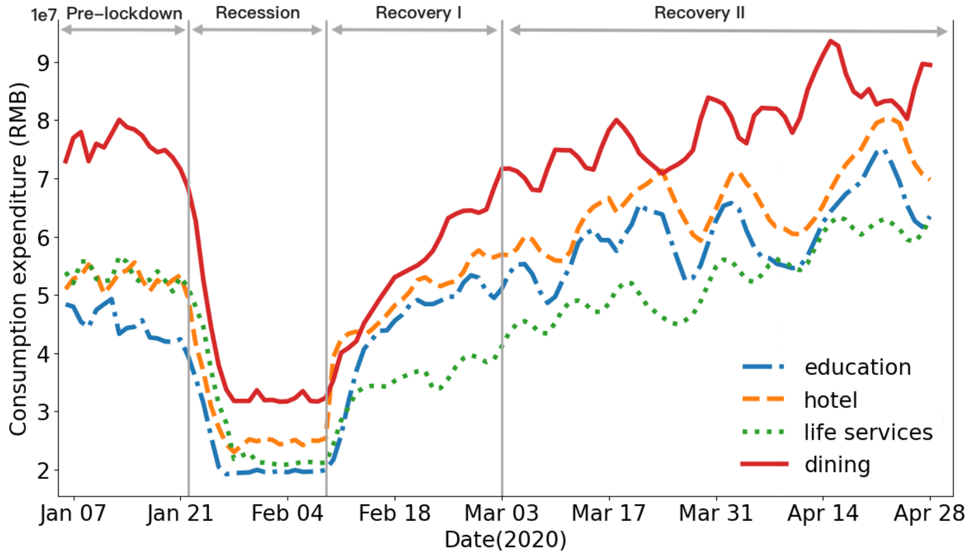

Supplementary Figure 1. Time series of various sectors of aggregate national consumption, which start to recover after February 10, 2020, when resumption of work and production is allowed (start of Recovery I).

Supplementary Note 3

Panel Regressions on Consumption in Each Phase

To dig deeper into the principal factors affecting city-level consumption in various phases, separate panel regressions for each phase (Recession, Recovery I and Recovery II) are performed. Note that, socioeconomic variables have varying degrees of impact on city-level consumption at different phases during the pandemic (Supplementary Table 1-6).

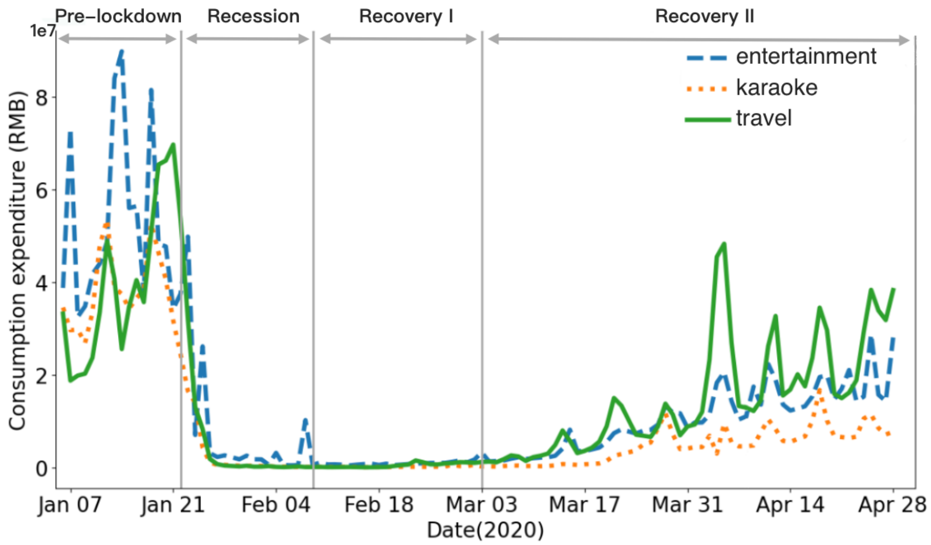

Supplementary Figure 2. Time series of leisure-related sectors of aggregate national consumption, which start to recover after March 3, 2020, when the gathering places reopen (start of Recovery II).

Based on the results of these panel regressions for both YGI and RGI, we can get some common insights. Income and GDP are the most impactful variables to understand the recession phase, while the economic structure contributes more to the degree of consumption recovery both for year-over-year growth index (YGI) and recovery gap index (RGI). Specifically, the secondary sector counts more during Recovery Phase I, and the tertiary sector is crucial to Recovery Phase II. The key result is that tertiary sector generate more impact in the later phase of consumption recovery than secondary sector does in the early phase.

In terms of panel regressions for RGI alone, income is the most relevant variable to understanding the consumption recovery gap: the coefficients of the income variable are substantially large in all phases of the regression (Supplementary Note 2 4 6). It makes sense that income will directly impact the living standards of citizens, which will have a significant impact on urban consumption. As a consequence, high-income groups usually consume more, resulting in a wider consumption gap due to the decrease in consumption during the pandemic.

Supplementary Note 4

Research Based on Consumption Orders

Our data allows us to measure the impact of the COVID-19 pandemic on consumption behaviors from two perspectives: the consumption expenditure as well as the volume of consumption orders. Previous analyses were all based on consumer expenditure to examine the change patterns of residents' consumption during the pandemic, including detailed change of consumption across cities and sectors. To further test the results obtained earlier, we additionally provide a study of consumption orders and obtain results consistent with the study based on consumption expenditures (Supplementary Figures 3-6).

As shown in Supplementary Figures 3(a) and 3(b), consumption orders of residents show an apparent v-shaped recession and recovery pattern with three distinct phases in terms of aggregate national statistics, which consistent with the trend of national consumption expenditure.

In Supplementary Figure 4(a), we investigate the recession and recovery rate of consumption orders in 53 cities. Cities with a larger number of infected COVID-19 cases saw a much faster rate of consumption decline, as the pandemic outbreak led to a significant drop in volume of consumption orders. Likewise, recession and recovery rates of consumption orders are linearly and positively correlated with one another. And the recovery rate is associated with differences in city-level economic structure (Supplementary Figure 4(b)).

We explore the dynamics of the share of consumption across sectors during the pandemic, both for the country and for cities with different economic structures. Then we observed similar change patterns of consumption structure in terms of consumption orders (Supplementary Figure 5 and 6).

Supplementary Note 5

Description of Data Sectors and Validity

The raw consumption records are shown in Supplementary Table 7. Residents' consumption expenditure refers to the regular and repeated consumption expenditure in the daily life of residents for the purpose of satisfying their own needs and those of

Supplementary Table 1. Panel regression of daily year-over-year growth index (YGI) of urban residents' consumption expenditure in recession phase. Column (2) add a interaction term between socioeconomic variables with nationwide infected cases of COVID-19. Column (3) controls for city fixed effects. Standard errors are clustered at the city level. Consumption data from January 6 to February 9, 2020 and the same period in 2019 (lunar calendar).

**Panel regression model for recession phase**			
**Dependent variable**			
Year-over-year growth index (YGI)			
**Independent variable**	(1)	(2)	(3)
Age between 16-59 (labor force)	0.175**	0.119**	0.103**
GDP	0.272***	0.267***	0.279***
Secondary sector	0.335***	0.326***	0.301***
Tertiary sector	0.234***	0.288***	0.256***
Income	0.295***	0.307***	0.31***
Infected cases of COVID	-0.073**	-0.092**	-0.103**
Nationwide infected cases of COVID	-0.387***	-0.399***	-0.386***
**Interaction term**			
Age between 16-59 (labor force)	N	0.006	0.014
GDP	N	-0.256**	-0.262**
Secondary sector	N	0.024***	0.032***
Tertiary sector	N	-0.117***	-0.109***
Income	N	-0.273***	-0.285***
City fixed effects	N	N	Y
R^2	0.487	0.579	0.647
***p $<$ 0.01; **p $<$ 0.05			

their family members. According to the residents' daily needs, the National Bureau of Statistics has divided the eight sectors of daily household expenditure into: leisure, life, living, dining, education, medicine, car, and others. In order to facilitate the study of the dynamics of the consumption structure, we divided the 18 sectors of consumption in Meituan into the eight sectors mentioned above. Auxiliary information on consumption sectors is also provided, including first-level sector name, second-level sector name, and proportion (Supplementary Table 8).

We next compare consumption data for each sector from Meituan with the national account household consumption series (Per Capita Expenditures on Consumption of Urban Households) for each corresponding sector in 2020. Specifically, we calculated the Pearson correlation coefficient between state-level consumption of our data and state-level national accounts household consumption for each quarter in 2020 from the temporal dimension, which is taken as the temporal correlation of consumption across each sector.

Due to the scarcity of national accounts data in the temporal dimension, we further verify our data in the spatial dimension. Likewise, we calculated the pearson correlation coefficient between city-level consumption of our data and city-level national accounts consumption in 2020 from the spatial dimension, taking them as the spatial correlation of consumption across each sector. The correlations are higher in the spatial dimension than in the temporal dimension, though all coefficient values are larger than 0.56 (Supplementary Table 8). This is probably explained by the more extensive data on national consumption at the city level, which allows us to obtain a more accurate correlation.

The conclusion is that our data captures important patterns across space and time in national accounts data, making it possible to act as an informative proxy along comparable cuts of official data.

Supplementary Note 6

Definition of Consumption Structure

Consumption Structure: The meaning of "consumption structure" is the relationship between the proportion of various consumption expenditures in a certain period. According to the different aspects of citizens' consumption expenditures, different forms of consumption structures can be formed, such as dining, education, living, and leisure. Here we focus on the consumption structure constituted by eight sectors of consumption expenditures that are most related to daily life.

National Consumption Structure: In our data, the fundamental units are city-level daily consumption expenditures. The

Supplementary Table 2. Panel regression of daily Recovery gap index (RGI) of urban residents' consumption expenditure in recession phase. Column (2) add a interaction term between socioeconomic variables with nationwide infected cases of COVID-19. Column (3) controls for city fixed effects. Standard errors are clustered at the city level. Consumption data from January 6 to February 9, 2020 and the same period in 2019 (lunar calendar).

**Panel regression model for recession phase**			
**Dependent variable**			
Recovery gap index (RGI)			
**Independent variable**	(1)	(2)	(3)
Age between 16-59 (labor force)	0.129**	0.142**	0.149**
GDP	0.208***	0.219***	0.229***
Secondary sector	0.183***	0.196***	0.187***
Tertiary sector	0.186***	0.192***	0.206***
Income	0.291***	0.296***	0.311***
Infected cases of COVID	0.014**	0.018**	0.021**
Nationwide infected cases of COVID	0.422***	0.424***	0.429***
**Interaction term**			
Age between 16-59 (labor force)	N	-0.039	-0.034
GDP	N	0.257**	0.261**
Secondary sector	N	-0.004***	-0.008***
Tertiary sector	N	0.103***	0.109***
Income	N	0.313***	0.328***
City fixed effects	N	N	Y
R^2	0.459	0.566	0.598
***p < 0.01; **p < 0.05			

meaning of the national consumption structure is that we aggregate daily consumption data over all cities to form the share of each consumption sector at the national level.

Supplementary Note 7

Definition of the secondary and tertiary sectors

According to the official statistical standards declared by the National Bureau of Statistics(https://data.stats.gov.cn), the division of economic structure is defined.

the secondary sector: The secondary sector is the sector that processes raw materials into material goods to meet the further needs of the citizens' production. It includes mining, manufacturing, electricity, heat, gas, and water production and supply, as well as the construction industry.

the tertiary sector: The tertiary sector refers to service-related industries that do not produce material goods. It contains two types of industries: one for production-oriented services, such as finance, software, and Internet service, and the other for serving citizens' demands, such as movies, sports, networking, games, animation, and so on.

Supplementary Table 3. Panel regression of daily year-over-year growth index (YGI) of urban residents' consumption expenditure in recovery phase I. Column (2) add a interaction term between socioeconomic variables with nationwide infected cases of COVID-19. Column (3) controls for city fixed effects. Standard errors are clustered at the city level. Consumption data from February 9 to March 3, 2020 and the same period in 2019 (lunar calendar).

**Panel regression model for recovery phase I**			
**Dependent variable**			
Year-over-year growth index (YGI)			
**Independent variable**	(1)	(2)	(3)
Age between 16-59 (labor force)	0.233**	0.209**	0.221**
GDP	0.206***	0.210***	0.227***
Secondary sector	0.285***	0.311***	0.326***
Tertiary sector	0.291***	0.299***	0.286***
Income	0.312***	0.363***	0.344***
Infected cases of COVID	-0.027**	-0.024**	-0.029**
Nationwide infected cases of COVID	-0.311***	-0.307***	-0.283***
**Interaction term**			
Age between 16-59 (labor force)	N	0.014	0.019
GDP	N	-0.122**	-0.102**
Secondary sector	N	0.420***	0.392***
Tertiary sector	N	-0.105***	-0.111***
Income	N	-0.183***	-0.177***
City fixed effects	N	N	Y
R^2	0.441	0.553	0.638
***p $<$ 0.01; **p $<$ 0.05			

Supplementary Table 4. Panel regression of daily Recovery gap index (RGI) of urban residents' consumption expenditure in recovery phase I. Column (2) add a interaction term between socioeconomic variables with nationwide infected cases of COVID-19. Column (3) controls for city fixed effects. Standard errors are clustered at the city level. Consumption data from February 9 to March 3, 2020 and the same period in 2019 (lunar calendar).

**Panel regression model for recovery phase I**			
**Dependent variable**			
Recovery gap index (RGI)			
**Independent variable**	(1)	(2)	(3)
Age between 16-59 (labor force)	-0.137**	0.141**	0.144**
GDP	-0.231***	-0.233***	-0.239***
Secondary sector	-0.122***	-233***	-0.239***
Tertiary sector	-0.188***	-0.191***	-0.203***
Income	-0.321***	-0.336***	-0.341***
Infected cases of COVID	0.045**	0.051**	0.058**
Nationwide infected cases of COVID	0.422***	0.424***	0.429***
**Interaction term**			
Age between 16-59 (labor force)	N	-0.025	-0.029
GDP	N	0.249**	0.241**
Secondary sector	N	-0.179***	-0.163***
Tertiary sector	N	0.151***	0.149***
Income	N	0.298***	0.287***
City fixed effects	N	N	Y
R^2	0.464	0.543	0.611
***p $<$ 0.01; **p $<$ 0.05			

Supplementary Table 5. Panel regression of daily year-over-year growth index (YGI) of urban residents' consumption expenditure in recovery phase II. Column (2) add a interaction term between socioeconomic variables with nationwide infected cases of COVID-19. Column (3) controls for city fixed effects. Standard errors are clustered at the city level. Consumption data from March 3 to April 28, 2020 and the same period in 2019 (lunar calendar).

**Panel regression model for recovery phase II**			
**Dependent variable**			
Year-over-year growth index (YGI)			
**Independent variable**	(1)	(2)	(3)
Age between 16-59 (labor force)	0.101**	0.009**	0.102**
GDP	0.215***	0.209***	0.210***
Secondary sector	0.254***	0.301***	0.308***
Tertiary sector	0.302***	0.297***	0.292***
Income	0.308***	0.323***	0.341***
Infected cases of COVID	-0.120**	-0.134**	-0.135**
Nationwide infected cases of COVID	-0.201***	-0.218***	-0.222***
**Interaction term**			
Age between 16-59 (labor force)	N	0.005	0.012
GDP	N	-0.253**	-0.210**
Secondary sector	N	0.131***	0.149***
Tertiary sector	N	-0.295***	-0.291***
Income	N	-0.222***	-0.231***
City fixed effects	N	N	Y
R^2	0.439	0.572	0.649
***$p < 0.01$; **$p < 0.05$			

Supplementary Table 6. Panel regression of daily Recovery gap index (RGI) of urban residents' consumption expenditure in recovery phase II. Column (2) add a interaction term between socioeconomic variables with nationwide infected cases of COVID-19. Column (3) controls for city fixed effects. Standard errors are clustered at the city level. Consumption data from March 3 to April 28, 2020 and the same period in 2019 (lunar calendar).

**Panel regression model for recovery phase II**			
**Dependent variable**			
Recovery gap index (RGI)			
**Independent variable**	(1)	(2)	(3)
Age between 16-59 (labor force)	-0.097**	0.111**	0.114**
GDP	-0.191***	-0.193***	-0.209***
Secondary sector	-0.117***	-122***	-0.138***
Tertiary sector	-0.179***	-0.182***	-0.198***
Income	-0.321***	-0.336***	-0.341***
Infected cases of COVID	0.158**	0.161**	0.167**
Nationwide infected cases of COVID	0.310***	0.304***	0.309***
**Interaction term**			
Age between 16-59 (labor force)	N	-0.022	-0.026
GDP	N	0.236**	0.239**
Secondary sector	N	-0.009***	-0.105***
Tertiary sector	N	0.179***	0.184***
Income	N	0.272***	0.274***
City fixed effects	N	N	Y
R^2	0.477	0.549	0.621
***$p < 0.01$; **$p < 0.05$			

Supplementary Table 7. Examples of consumption records. Note that city is labeled with **City ID**, consumption date is labeled with **Date**, consumption sector is labeled with **Sector ID**, the volume of consumption orders is labeled with **Order**, and the consumers' expense is labeled with **Expenditure**.

City ID	Date	Sector ID	Order	Expenditure
1	2020/3/1	1853	2625	365851.06
1	2020/3/1	2	5897	953851.5
1	2020/3/1	389	503	87097.98
1	2020/3/1	3	6099	1464980.53

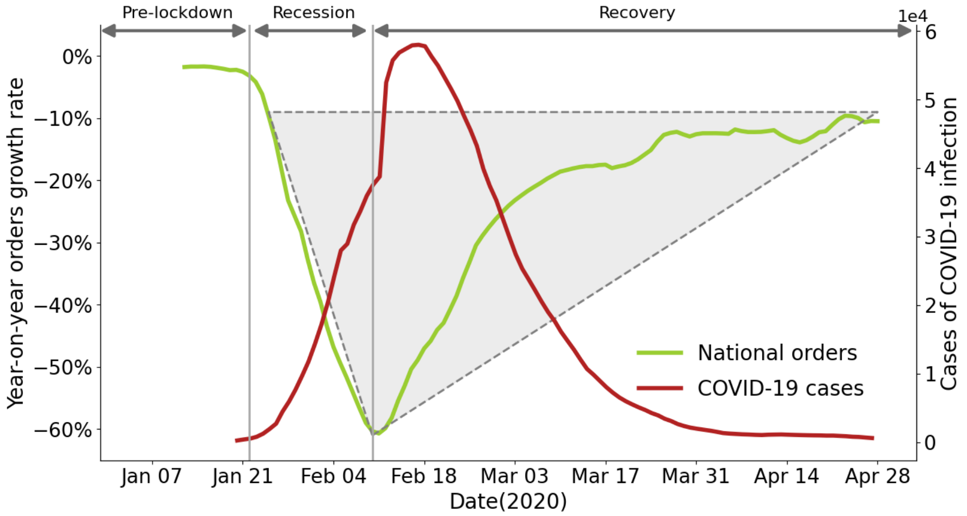

(a) Y-o-Y growth of aggregate national consumption orders and confirmed cases of COVID-19 infection

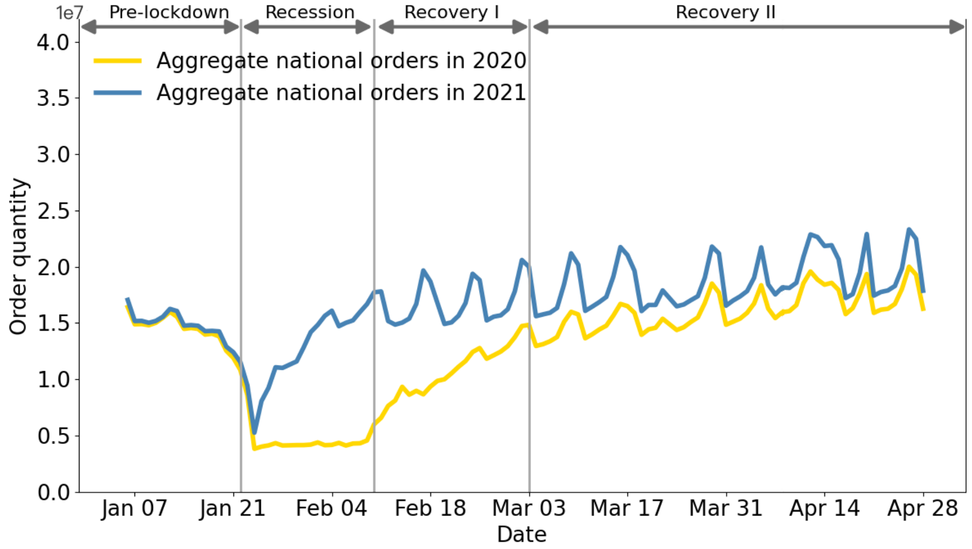

(b) Time series of aggregate national consumption orders

Supplementary Figure 3. Temporal variations of aggregate national consumption orders during COVID-19 pandemic. (a) Moving average (7 days, uncentred) of year-over-year growth rate of aggregate national consumption orders and cases of confirmed COVID-19 infection (b) Time series of aggregate national consumption orders in 2020 and 2021.

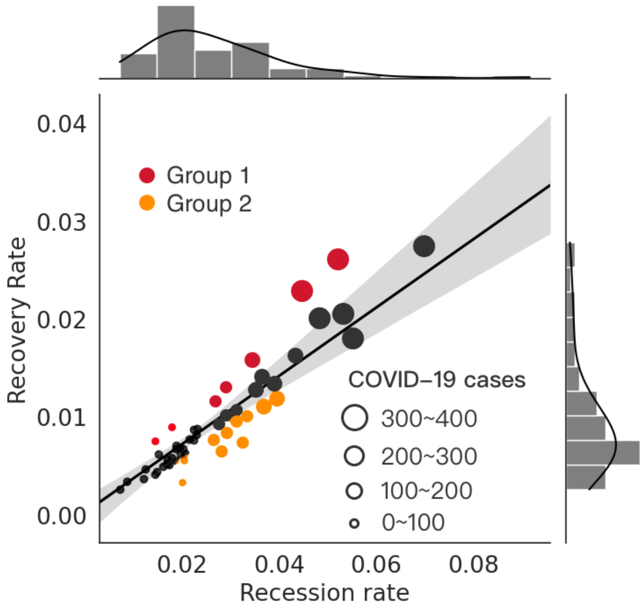

(a) Scatter plot of rate

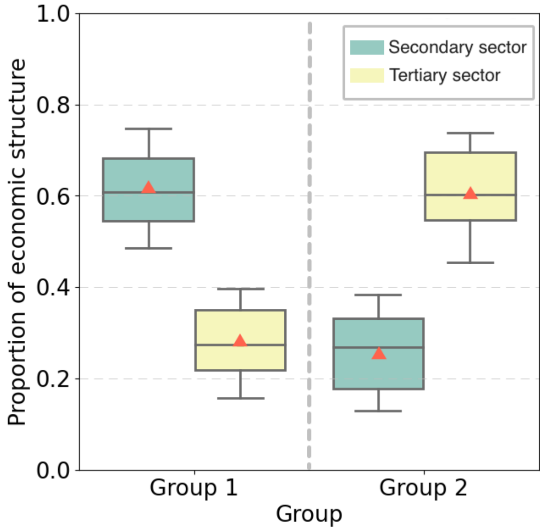

(b) Distribution across groups

Supplementary Figure 4. Recession and recovery pattern of consumption orders for 53 cities.

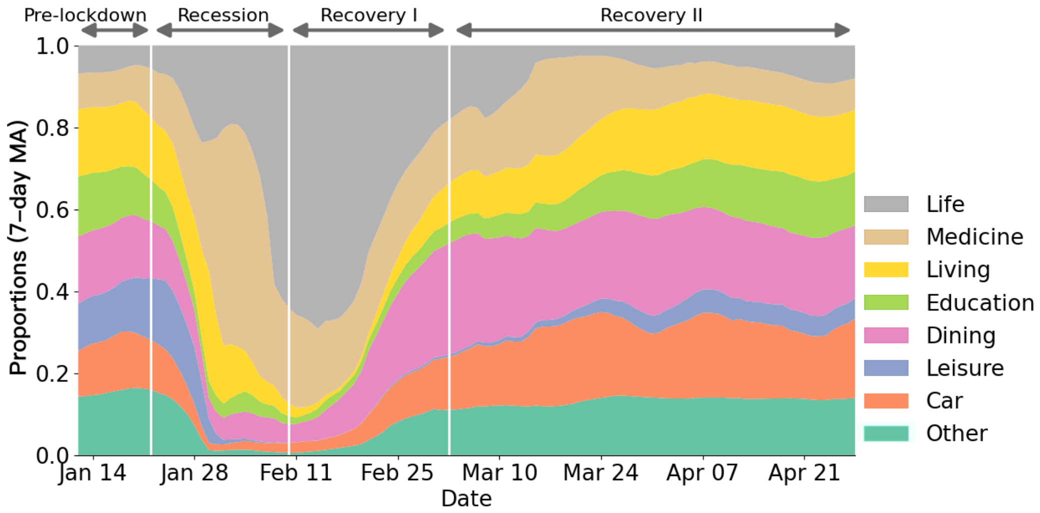

Supplementary Figure 5. Evolution of the share of aggregate national consumption orders across categories during COVID-19 pandemic.

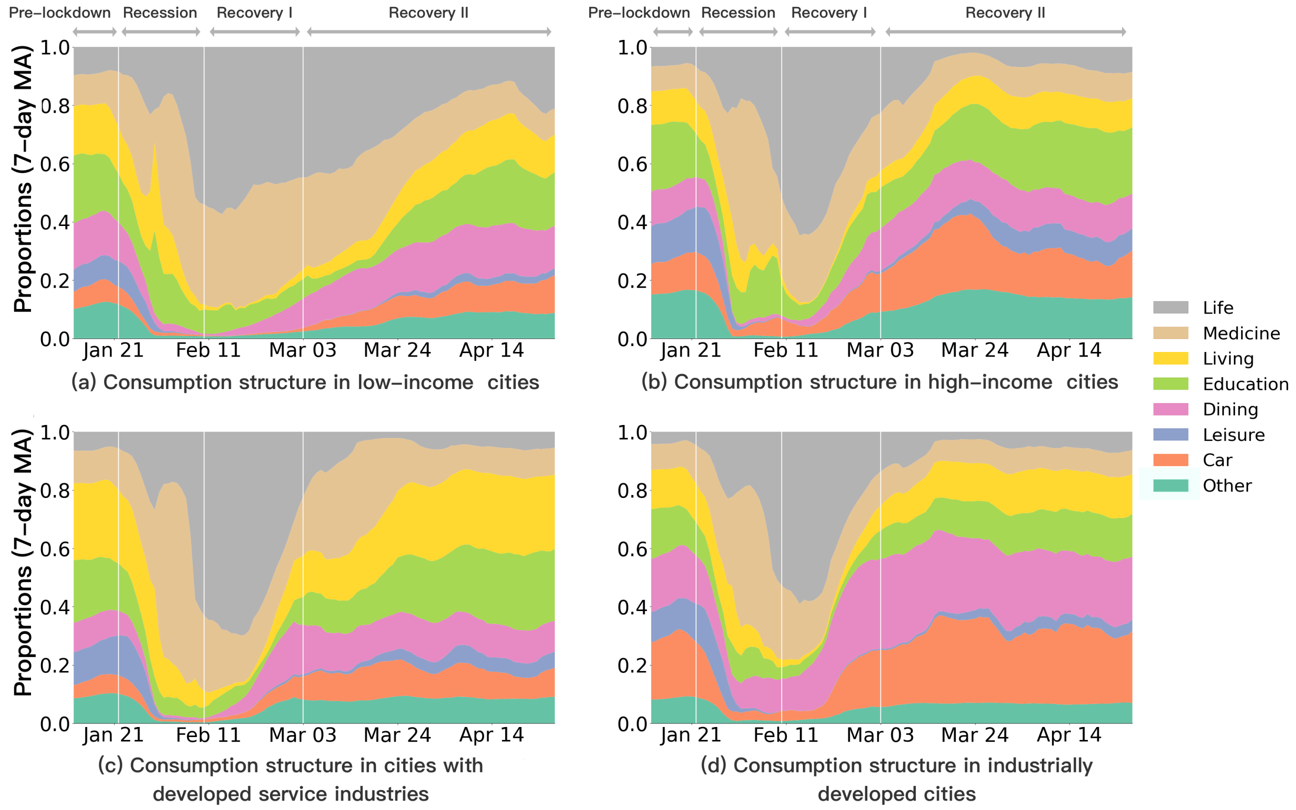

Supplementary Figure 6. Dynamics of city-level consumption orders during COVID-19 pandemic.

Supplementary Table 8. Dataset. Statistics about consumption sectors in this study and their correlation with national accounts.

First-level sector	Second-level sector	Proportion	Temporal correlation	Spatial correlation
Leisure	Entertainment	0.0028	0.7162	0.8233
	Sports	0.0113		
	Travel	0.0679		
	Karaoke	0.0590		
	Movie	0.0821		
Life	Life services	0.0692	0.8244	0.8454
	Households	0.0122		
	Shopping	0.0432		
	Wedding	0.0325		
	Offspring	0.0387		
Living	Hotel	0.1970	0.6547	0.8123
	Homestay	0.0178		
Dining	Dining	0.2415	0.6997	0.7986
Education	Education	0.0248	0.5842	0.7877
Medicine	Medicine	0.0404	0.6317	0.7953
Car	Car	0.0041	0.5865	0.6221
Other	Pet	0.0019	0.5613	0.6189
	Beauty	0.0494		
